# Supplementary material for: Knockout of Perilipin-2 in Microglia Alters Lipid Droplet Accumulation and Response to Alzheimer’s Disease Stimuli
Source: Cells. 2025 Nov 13;14(22):1783. doi: 10.3390/cells14221783 (PMC12651832; doi:10.3390/cells14221783)
Supplement: Supplementary file 1 [file cells-14-01783-s001.zip › cells-3919728- Supplementary Figures CONVERSION 11.13.pdf]

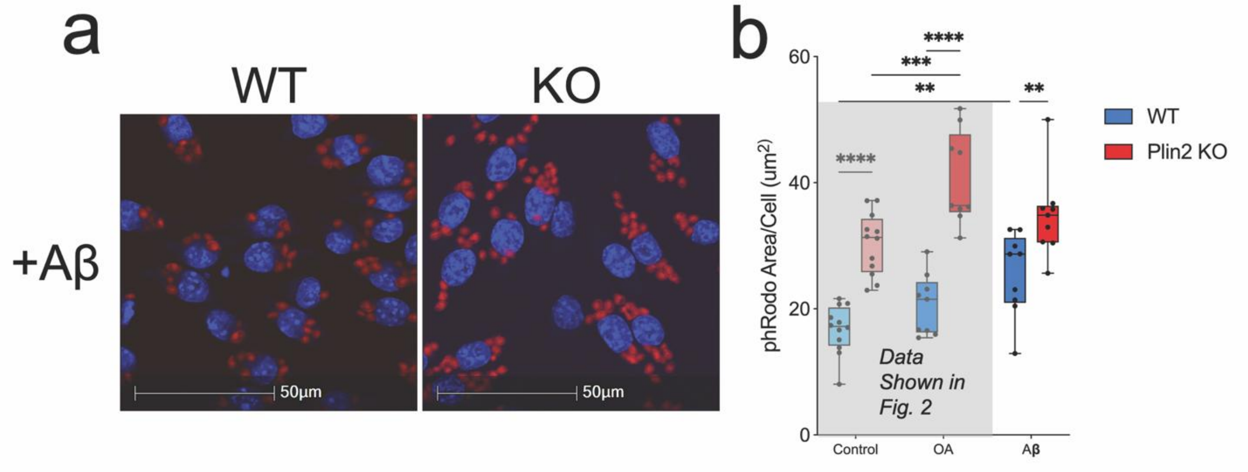

Supplementary Figure S1: Plin2 loss enhances phagocytosis in BV2 microglia.
(a) Representative pHrodo™ Red Zymosan images under Aβ challenge; nuclei (DAPI, blue). Scale bars, 50 μm. **(b)** Quantification of pHrodo area per cell for Control, OA, and Aβ in WT (blue) and Plin2-KO (red). Points = wells (n = 6 per condition). Two-way ANOVA (Genotype × Condition) with Sidak post-hoc; $^{*}p < 0.05$, $^{**}p < 0.01$, $^{***}p < 0.001$, $^{****}p < 0.0001$. KO shows higher uptake than WT across conditions.

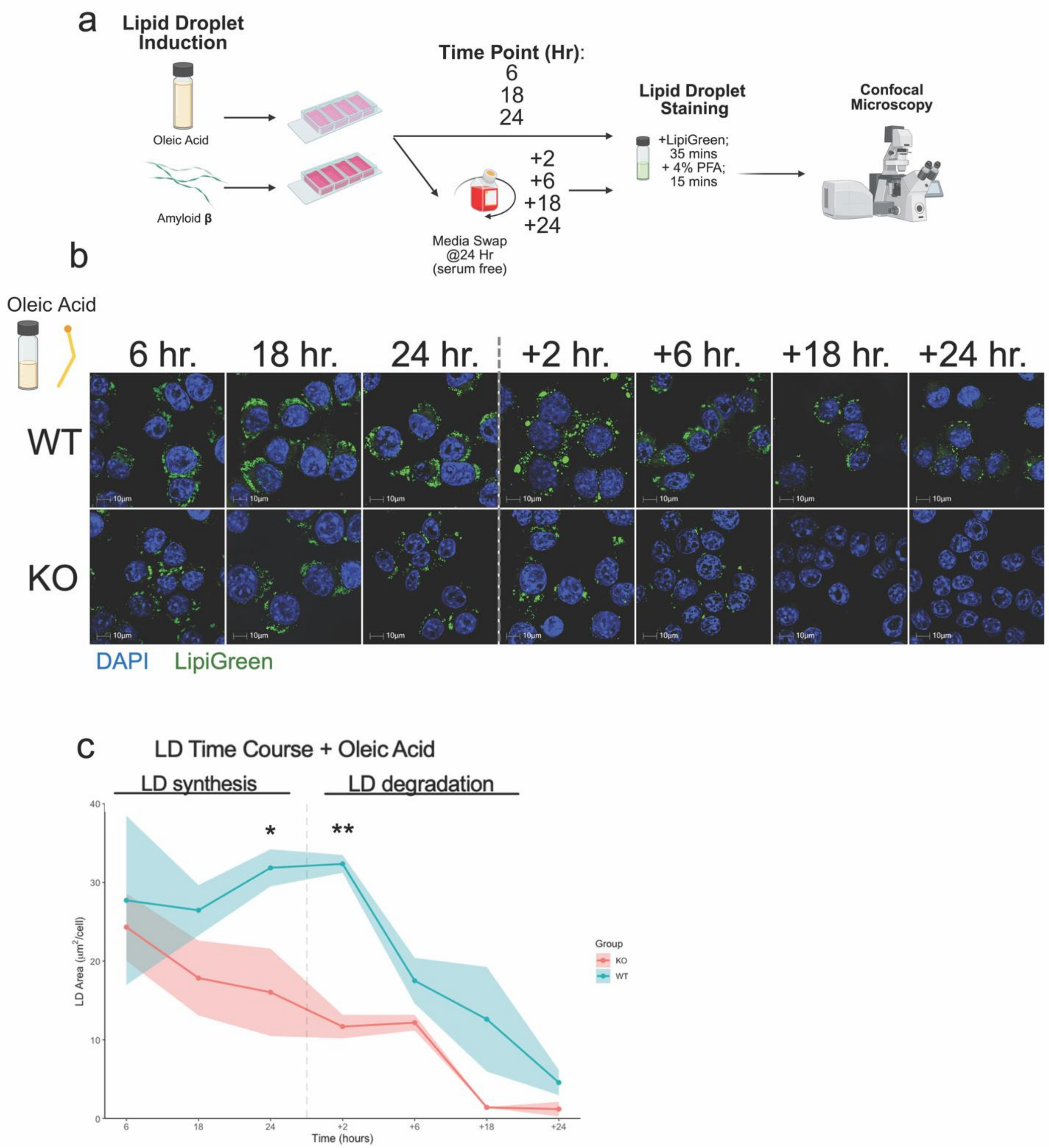

Supplementary Figure S2. Oleic-acid (OA) time course in BV2 microglia.
(a) Schematic of the assay: cells were exposed to OA and imaged at 6, 18, and 24 h, then OA was removed (media swap) and cells were imaged at +2, +6, +18, and +24 h. Lipid droplets were labeled with Lipi-Green and nuclei with DAPI; confocal imaging. (b) Representative fields of OA treated WT and Plin2 KO at the indicated time points. Scale bar, 10 µm. (c) Total lipid-droplet area per nucleus across the time course (mean ± SEM). Statistics: two-way ANOVA (genotype × time) within treatment with Šídák post-hoc adjustment

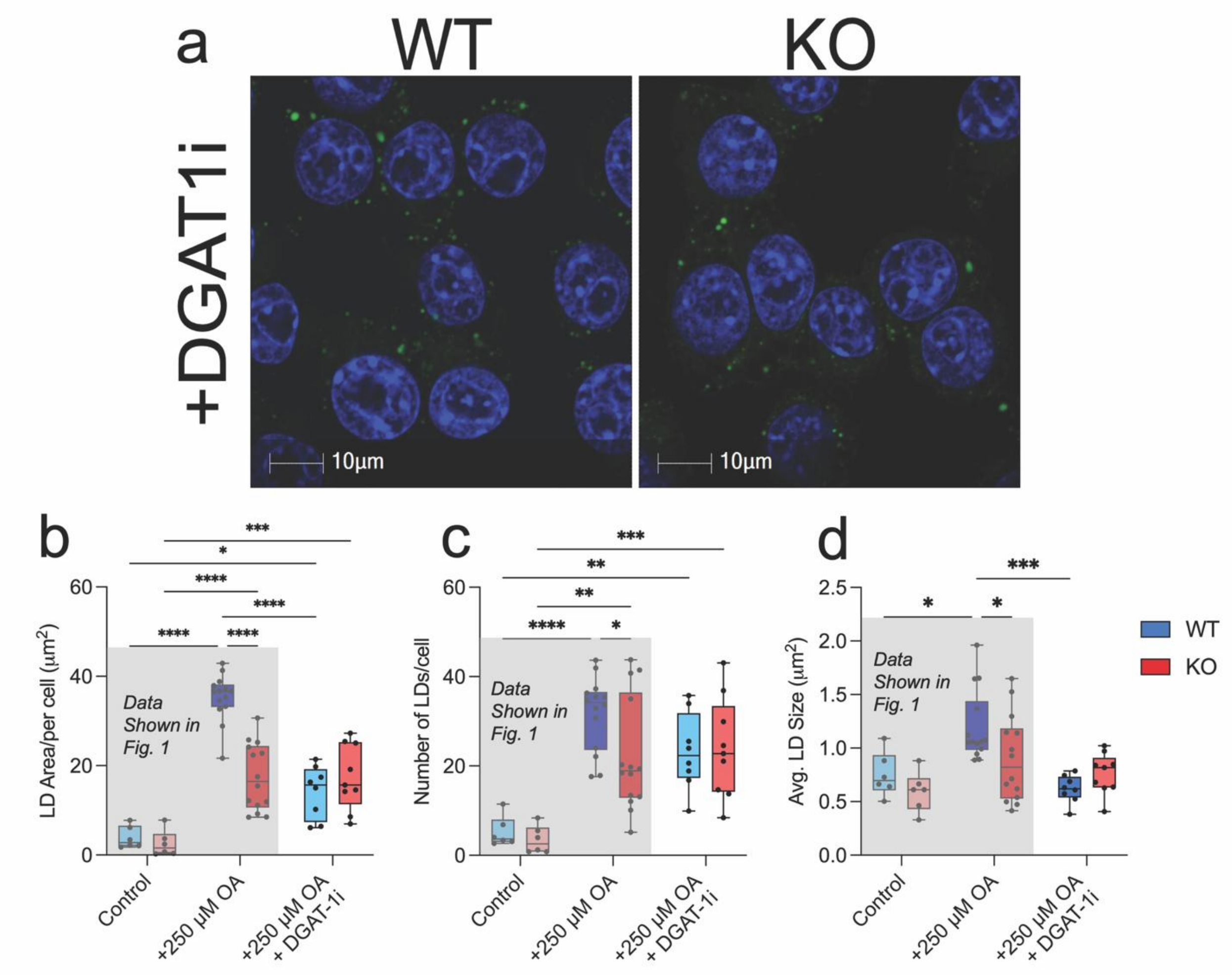

Supplementary Figure S3: DGAT1 inhibition limits OA-induced lipid droplets in BV2 microglia. (a) Confocal images after 2 h pre-treatment with A922500 (1.5 µM) and 18 h OA (250 µM); LipiGreen (green), DAPI (blue). Scale, 10 µm. (b–d) Quantification of total LD area/cell, LD number/cell, and mean LD size for Control, OA, and OA + DGAT1i in WT (blue) and Plin2-KO (red). Two-way ANOVA (GenotypexTreatment) with Sidak post-hoc; *p<0.05, **p<0.01, ***p<0.001, ****p<0.0001. n=9 wells/group .DGAT1 inhibition strongly blunts OA-induced LD load in WT and has a smaller effect in KO, indicating Plin2-dependent, DGAT-mediated TG storage.
